# Dehiscent Prosthetic Aortic Valve and Aortic Root Pseudoaneurysm Complicated by Left Main Coronary Artery Compression

**DOI:** 10.7759/cureus.35096

**Published:** 2023-02-17

**Authors:** Ebubechukwu Ezeh, Maddie Perdoncin, Mohamed Suliman, Esiemoghie J Akhigbe, Rameez Sayyed

**Affiliations:** 1 Internal Medicine, Marshall University Joan C. Edwards School of Medicine, Huntington, USA; 2 Internal Medicine-Pediatrics, Marshall University Joan C. Edwards School of Medicine, Huntington, USA

**Keywords:** coronary artery compression, prosthetic heart valve, left main coronary artery disease, prosthetic valve dehiscence, aortic pseudoaneurysm

## Abstract

Prosthetic aortic valve dehiscence is an uncommon complication of prosthetic valve endocarditis that may occur in patients who have undergone aortic valve replacement (AVR). The concurrent presence of aortic root pseudoaneurysm may further complicate the clinical presentation through the external compression of coronary arteries. Thus, patients may present with clinical features of coronary ischemia. Echocardiogram and coronary angiography are useful in establishing diagnosis. Treatment involves a multidisciplinary approach involving cardiologists, infectious disease specialists, and cardiothoracic surgeons. The authors of this study discuss a 51-year-old male who presented with anginal chest pain and was found to have a new left bundle branch block, elevated troponins, and left main coronary artery compression complicating aortic root aneurysm. He ended up requiring a re-do AVR, repair of the pseudoaneurysm, and coronary artery bypass graft.

## Introduction

Prosthetic aortic valve infection with dehiscence is a major complication in patients with prior valve replacements [1]. This complication occurs in approximately 0.1% to 1.3% of aortic valve replacement surgeries and is more common in patients with pre-existing bacterial endocarditis and severe calcification of the aortic valve [1]. Dehiscence may be accompanied by external compression of coronary circulation by an aortic root aneurysm [2]. Such an external compression of coronary arteries is a rare cause of myocardial ischemia [2]. Presenting symptoms in these patients can therefore vary widely. Auscultatory findings will usually suggest valve insufficiency, but ultimately the severity of the underlying pathology would be defined by echocardiographic and angiographic findings. In this report, the authors discuss a case of aortic root aneurysm and left main coronary artery compression in a 51-year-old male with a dehiscent prosthetic aortic valve.

## Case presentation

A 51-year-old male was referred from an outside facility on account of dyspnea, anginal chest discomfort and elevated troponins. His medical history was significant for intravenous (IV) drug use, hepatitis C, and multiple cerebrovascular accidents (CVAs) that necessitated patent foramen ovale closure. His cardiac history was significant for infective endocarditis-related valve dysfunctions status-post bio-prosthetic aortic valve replacement (AVR) and mitral valve repair with an annuloplasty ring.

**Figure 1:**
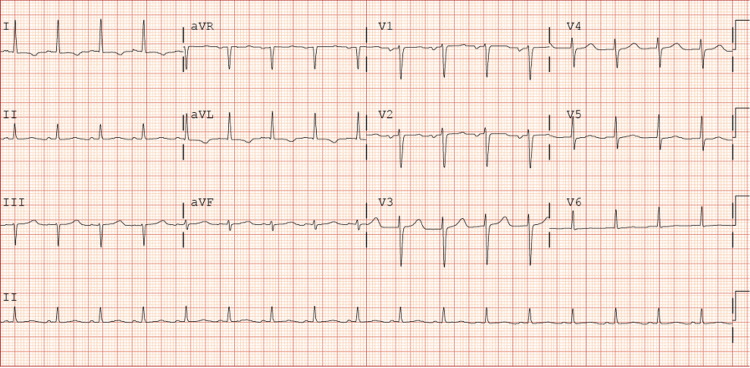
Electrocardiogram obtained three months prior to the presentation. The electrocardiogram is showing sinus rhythm with no left bundle branch block.

**Figure 2:**
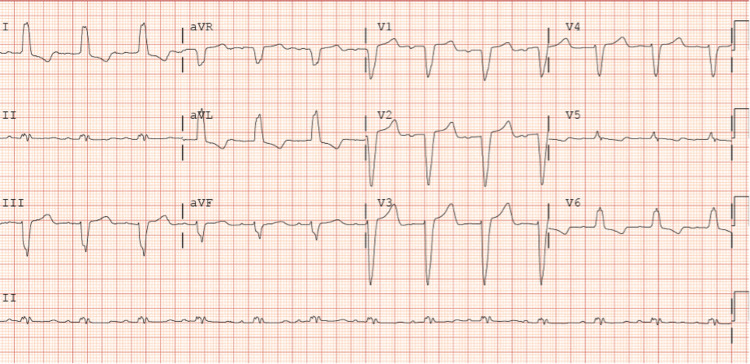
Electrocardiogram obtained on admission. The electrocardiogram is showing sinus rhythm and a new left bundle branch block.

His vital signs included a blood pressure of 130/74 millimeters of Mercury, a pulse rate of 86 beats/minute, and a temperature of 98.6 degrees Fahrenheit. Examination findings were notable for crackles on lung auscultation. Initial lab results showed an elevated high-sensitivity troponin I level of 327 picogram per milliliter (pg/ml) (reference range 3-58 pg/ml). Rapid plasma reagin was negative. Computed tomography (CT) of the lungs suggested pulmonary septic emboli. Electrocardiogram (ECG) showed sinus rhythm and a new left bundle branch block (LBBB) that was not present on an ECG obtained three months earlier. Both ECGs are shown in Figures 1-2.

**Figure 3:**
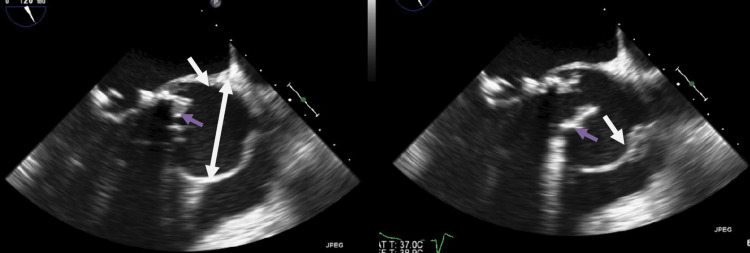
Transesophageal echocardiogram. Blue arrows show the prosthetic valve and white arrows show the walls of the aortic root pseudoaneurysm.

**Figure 4:**
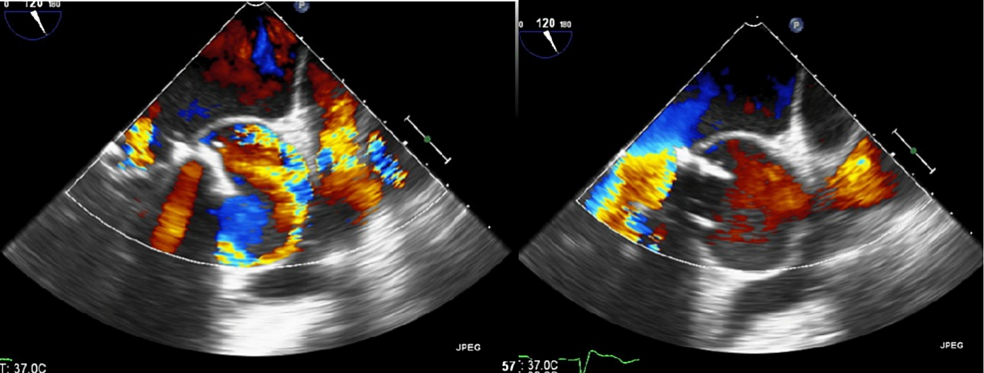
Transesophageal echocardiogram. The images are showing regurgitation through the prosthetic aortic valve and aortic root pseudoaneurysm.

The cardiology service was consulted. A transthoracic echocardiogram (TTE) was performed and showed an abnormal prosthetic aortic valve function with rocking motion, prosthesis stenosis with peri-valvular regurgitation, pseudo-aneurysm of the aortic root, and ejection fraction of 25%. A transesophageal echocardiogram (TEE) was pursued and showed prosthesis stenosis, high sense of aortic valve more than 180 degrees detached with severe perivalvular regurgitation, and sewing ring dehiscence in the prosthetic aortic valve. It also showed a 4.7 cm by 4.3 cm aortic root pseudoaneurysm communicating with the aortic sinus (Figures 3-4).

**Figure 5:**
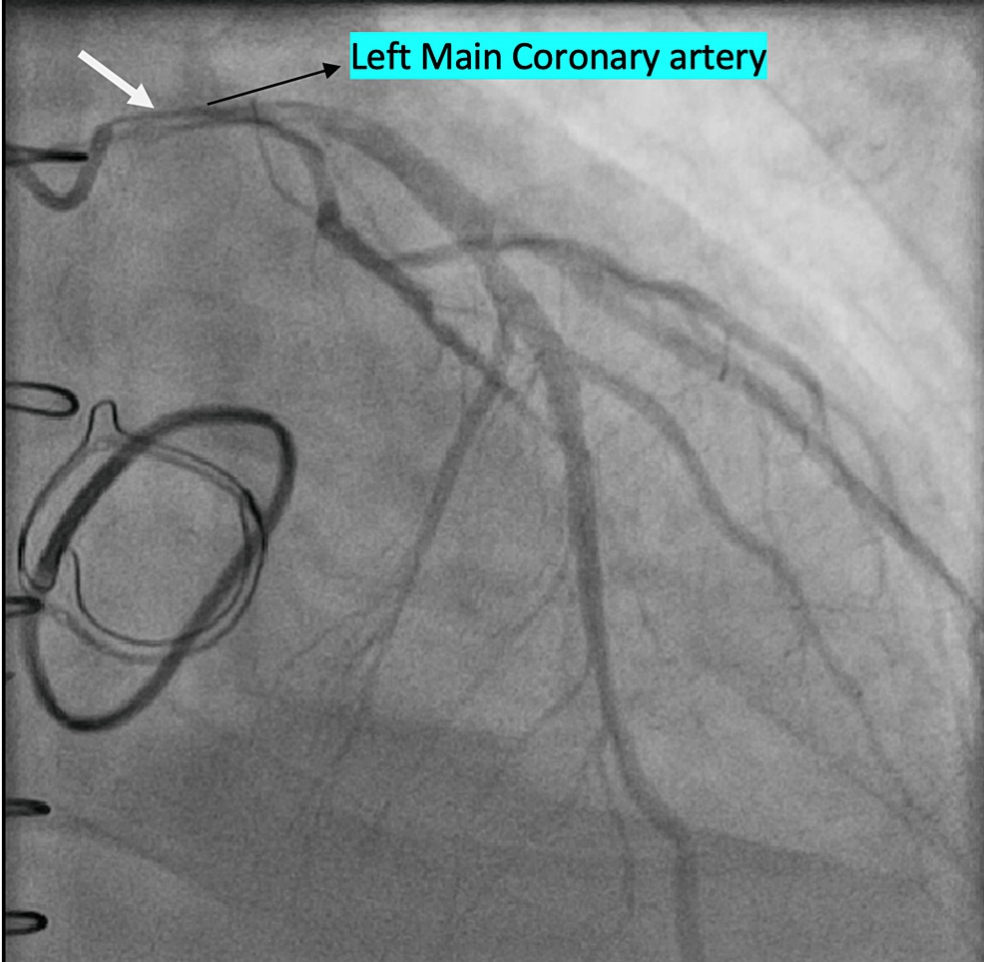
Coronary angiogram. The white arrow is pointing to the area of compression of the left main coronary artery.

Though blood cultures remained negative, the pseudoaneurysm was believed to have a mycotic origin. Furthermore, the patient received antibiotics prior to his presentation. Left heart catheterization (LHC) was subsequently performed and showed external compression of the left main coronary artery (LMCA) and proximal left circumflex artery from the pseudoaneurysm as shown in Figure 5. The cardiothoracic surgery team was immediately consulted. The patient had a median sternotomy with the removal of vegetation from the prosthetic valve and the annulus. Repair of the aortic root pseudoaneurysm and a redo AVR with aortic root heart valve bioprosthesis 29 mm were performed.

**Figure 6:**
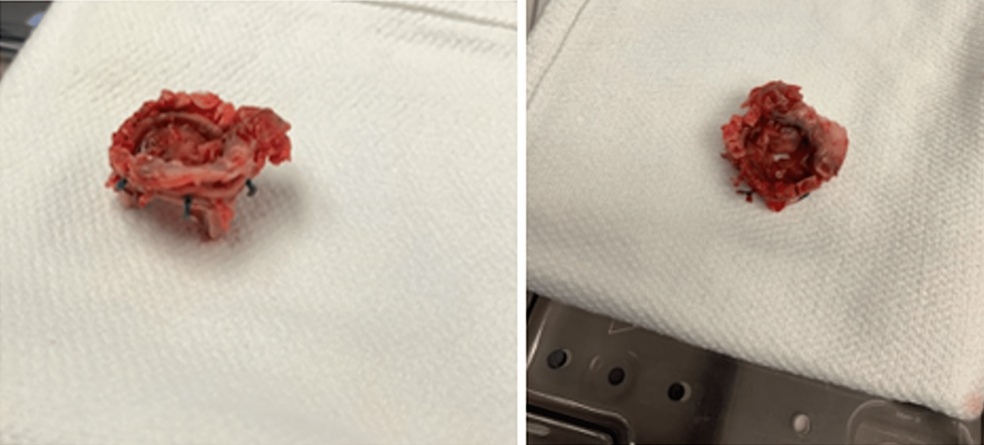
The dehiscent aortic valve.

The aortic root pseudoaneurysm was believed to be the cause of the external pressure on the LMCA as demonstrated in the coronary angiogram; coronary artery bypass graft (CABG) was thus not planned initially. The plan, however, changed during the aneurysmal repair when the patient developed elevated pulmonary artery (PA) pressure and a visibly distended heart. CABG with saphenous vein graft to the left anterior descending artery was therefore performed. PA pressure normalized after the CABG. The dehiscent aortic valve is shown in Figure 6. Pathologically, the aortic root aneurysm showed vascular wall tissue with degenerative changes with few mixed inflammatory cells. A cardiac resynchronization therapy-defibrillator (CRT-D) device was placed on account of severe cardiomyopathy. Follow-up limited echo showed normal functioning aortic valve prosthesis post median sternotomy and post-CABG TEE showed tissue valve in aortic position with no leak. The patient’s symptoms gradually improved. He was placed on long-term antibiotics, made an uneventful recovery, and was discharged to cardiac rehabilitation.

## Discussion

Unlike an atherosclerotic disease, external compression of the LMCA is a much less common cause of coronary ischemia. Etiologies include pulmonary artery in the setting of primary pulmonary hypertension, perivalvular abscess of the aortic valve, aneurysm of the aortic root, and left ventricular aneurysm [2-4]. Patients may be asymptomatic or present with clinical evidence of ventricular ischemia [5]. The present case had compression of LMCA by a large aortic root pseudoaneurysm and presented with chest pain and dyspnea. The incidence of extrinsic coronary compression complicating aortic root pathologies was thought to be 40% in a large autopsy series with endocarditis [6]. Negative rapid plasma reagin (RPR) made syphilitic aortic disease unlikely in the present case. Other rare etiologies include a noninfectious inflammatory vasculitis such as Takayasu disease for which our patient did not meet the diagnostic criteria [7].

Prosthetic valve endocarditis (PVE) can result in valve dehiscence and mycotic aortic root aneurysm [1]. The incidence of prosthetic aortic valve dehiscence post AVR is reported to be 0.1-1.3% [1]. Despite his negative blood cultures, our patient had probable infective endocarditis by Duke’s criteria; thus, his aortic root pseudoaneurysm was believed to be mycotic in origin. The pathophysiologic basis of native and prosthetic valve dehiscence is different [8]. In native valves, ﻿destruction starts on the cusps where it results in early detectable regurgitation jets and paravalvular abscesses are less common [8]. In prosthetic aortic valves, the infection originates more frequently from the annulus, thereby causing separation of the prosthetic valve from the infected tissue with paravalvular leakage and sometimes a ‘‘rocking motion’’ of the partially detached aortic valve prosthesis [8]. The current case revealed a bioprosthetic valve and subsequent paravalvular leakage.

The diagnosis is usually made using echocardiography. It is important to remember that a negative TEE does not rule out the presence of vegetation. Large vegetation is a later event and the detection of smaller vegetation by a transesophageal echocardiogram (TEE) can be challenging due to the shadowing effect and reflection of the prosthetic material [8]. Moreover, early prosthetic valve endocarditis (PVE) involves the junction of the sewing ring and annulus rather than the leaflets alone, leading to valve dehiscence and paravalvular leak [9]. As in the present study, TEE will usually demonstrate prosthesis stenosis with severe perivalvular regurgitation as well as sewing ring dehiscence.

The current case faced a distinctive complication involving compression of the left main coronary artery and proximal left circumflex artery by the aortic root pseudoaneurysm. ECG showed LBBB which was concerning for acute coronary syndrome necessitating coronary angiogram. LHC will usually show the area of external compression of the LMCA like in our patient. Surgical treatment of the aneurysms relieves the mechanical compression of the coronary arteries. Surgical management for PVE is not without challenges with some series reporting mortality rates of 20-60% and infection recurrence rate of about 7% [10]. Long-term antibiotic therapy is also indicated in cases of PVE. Our patient required aneurysmal repair, CABG, and antibiotics.

## Conclusions

Aortic root aneurysm causing external compression of LCA is an important but unusual cause of myocardial ischemia. Patients may be asymptomatic or present with chest pain and dyspnea. Management requires a multi-disciplinary approach. Since the clinical presentation is varied, clinicians need to be aware of the various presentations and challenge of variation in anatomic structure observed in management of such patients. Therefore, this is an important differential diagnosis to consider in the right clinical scenario: patients with PVE and aneurysmal aortic root, especially those with minimal to no atherosclerotic risk factors.
